# Old Receptor, New Tricks—The Ever-Expanding Universe of Aryl Hydrocarbon Receptor Functions. Report from the 4th AHR Meeting, 29–31 August 2018 in Paris, France

**DOI:** 10.3390/ijms19113603

**Published:** 2018-11-15

**Authors:** Charlotte Esser, B. Paige Lawrence, David H. Sherr, Gary H. Perdew, Alvaro Puga, Robert Barouki, Xavier Coumoul

**Affiliations:** 1 IUF-Leibniz Research Institute for Environmental Medicine, Auf´m Hennekamp 50, 40225 Düsseldorf, Germany; 2 Environmental Medicine, University of Rochester School of Medicine and Dentistry, 601 Elmwood Ave, Rochester, NY 14642, USA; Paige_Lawrence@URMC.Rochester.edu; 3 Department of Environmental Health, Boston University School of Public Health, 72 East Concord Street, Boston, MA 02118, USA; dsherr@bu.edu; 4 Center for Molecular Toxicology and Carcinogenesis, Department of Veterinary and Biomedical Sciences, The Pennsylvania State University, University Park, PA 16802, USA; ghp2@psu.edu; 5 Department of Environmental Health, University of Cincinnati College of Medicine, Cincinnati, OH 45267, USA; PUGAA@ucmail.uc.edu; 6 Toxicologie Pharmacologie et Signalisation Cellulaire, INSERM UMR-S1124, 45 rue des Saints-Pères, 75006 Paris, France; robert.barouki@parisdescartes.fr (R.B.); xavier.coumoul@parisdescartes.fr (X.C.); 7 UFR des Sciences Fondamentales et Biomédicales, Université Paris Descartes, 45 rue des Saints-Pères, Sorbonne Paris Cité, 75006 Paris, France

**Keywords:** transcription factor, translational science, environmental health, barrier organs, stem cells, nervous system, obesity, cancer, immunity, development, diet

## Abstract

In a time where “translational” science has become a mantra in the biomedical field, it is reassuring when years of research into a biological phenomenon suddenly points towards novel prevention or therapeutic approaches to disease, thereby demonstrating once again that basic science and translational science are intimately linked. The studies on the aryl hydrocarbon receptor (AHR) discussed here provide a perfect example of how years of basic toxicological research on a molecule, whose normal physiological function remained a mystery for so long, has now yielded a treasure trove of actionable information on the development of targeted therapeutics. Examples are autoimmunity, metabolic imbalance, inflammatory skin and gastro-intestinal diseases, cancer, development and perhaps ageing. Indeed, the AHR field no longer asks, “What does this receptor do in the absence of xenobiotics?” It now asks, “What doesn’t this receptor do?”.

## 1. Overview

The aryl hydrocarbon receptor (AHR), discovered 40 years ago, emerged initially as a novel class of protein: An orphan receptor that also acts as a transcription factor, mediating the toxicity of chemicals from the environment. However, our understanding of the scope of biological and pathophysiological processes, which AHR modulates, continues to expand, revealing that this receptor plays a central role in many disease conditions and thereby may hold the key to preventing and treating a broad range of maladies.

Many examples of AHR’s role in normal physiology and disease were presented at the 4th international meeting on AHR. Organized by Robert Barouki and Xavier Coumoul from the Université Paris Descartes, the meeting took place in Paris, France in late August, 2018. The first AHR-centered meeting in 2005 in Düsseldorf, Germany, focused strongly on the toxicology mediated by this ligand-activated receptor, which some still call the “dioxin-receptor”. However, new evidence for physiological functions emerged, first for the immune system and later for the nervous system, maintenance of barrier organs (e.g., skin and gut), metabolic regulation, host-microbiome interactions, and cancer progression. The meeting in Paris addressed these AHR-regulated processes, highlighted many more, and added a focus on the therapeutic opportunities arising from manipulating the AHR-signaling system. A major conclusion of the meeting was that this is the time to begin to apply our knowledge of AHR signaling to generate novel AHR-targeted therapeutics, a conclusion reinforced by the several pharmaceutical companies presenting their data at the meeting. That said, there is still much to learn about the AHR and its highly complex and tissue-specific functions.

### Biochemistry and Evolution of AHR

What is the AHR and why does it warrant a meeting dedicated solely to its function? The AHR has been called a link to the environment, a quorum sensor of bacteria, a pattern recognition receptor for chemicals, the mediator of the toxicity of polycyclic aromatic hydrocarbons (PAHs), and an attractive therapeutic target for cancer and immune diseases. These entire functions link AHR´s ability to bind a variety of ligands, frequently present as mixtures, with complex temporal patterns of regulation. With so many possible outcomes of AHR activation, it is critical that we learn more about what regulates AHR activity and what directs its biological outcomes.

Biochemically, AHR is a cytoplasm-located transcription factor, which shuttles to the nucleus upon binding of a range of small molecular weight molecules roughly the size of three benzene rings. These can be environmental chemicals such as dioxins or planar biphenyls or chemicals, plant-derived molecules such as flavonoids or indigo or endogenous compounds, such as tryptophan-derived compounds. Some authors classify ligands according to binding strength and persistence, others according to the source (xenobiotic versus natural) or to the health outcome (beneficial versus adverse). In any case, ligands are numerous and possibly the diversity of functions mediated by AHR-activation is driven by evolutionary selection processes. The AHR induces complex transcription patterns, as evidenced by RNAseq and microarray data using different cells or ligands. Many speakers at the meeting noted that transcription depends on the cellular context and interference or interaction with other cellular signaling pathways, e.g., inflammation. In any case, it is clear that AHR links the sensing of specific chemical signatures to critical body functions. While the study of the complexity of AHR signaling and its effect on biological systems was initially the purview of toxicologists, the connection now intrigues those studying developmental biology, immunology, various organ physiologies and resilience against stress, metabolism, cancer and those in search of valuable targets for prevention and therapy of a range of hard-to-treat diseases [1,2,3,4,5,6,7,8,9].

## 2. Sessions and Presentations

In 10 lecture sessions and a busy poster session, the meeting looked at AHR signaling during development, its effect on basic functions, and at its involvement in metabolic disease, cancer, and immune-mediated diseases. Pedro Fernandez-Salguero from the Universidad de Extremadura in Spain highlighted the role of the AHR in the balance of pluripotency and differentiation by stem cells using various tissues (i.e., lung, liver and hair). He found that the AHR, in that context, improves regeneration but inhibits cell differentiation. With elegant kinetic AHR knockdown experiments, he could demonstrate that the AHR is only needed at the start of re-differentiation, i.e., for the activation of stem cells [10,11]. The underlying mechanisms involve pluripotency factors such as the well-recognized octamer-binding transcription factor 4 (OCT-4) or the homeobox protein NANOG. In addition, the AHR is necessary to prevent over-active stem cell differentiation, which may lead to tumorigenesis. Indeed, expression of the reprogramming OCT-KLF4-SOX2-MYC2 transgene in an AHR-deficient background reduced mice survival of mice and promoted growth of undifferentiated teratomas. In the line of this work, Chia-I Ko from the University of Cincinnati showed that AHR deficiency in blastocysts leads to an increased number of cells. In keeping with the sometimes-contradictory results obtained in different systems, David Sherr (Boston University) discussed how the AHR, in several cancers including prostate, triple negative breast, and oral squamous cell cancer, appeared to drive stem cancer cell production and aggression. Another talk, given by Sylvie Bortoli (Paris Descartes University and INSERM), showed that AHR activity changes in the energy metabolism of tumor cells via mitochondrial dysfunction (from the respiratory chain), adding to the non-genotoxic involvement of AHR in tumors. Thomas Haarmann-Stemmann from the IUF in Düsseldorf, Germany, showed that the AHR represses nucleotide excision repair and apoptosis in UVB-irradiated keratinocytes. AHR-deficient mice were largely protected against UVB-induced skin photocarcinogenesis. Furthermore, the data revealed that the accelerated repair in AHR-compromised keratinocytes depended on a modulation of the tumor suppressor protein p27 [12]. The findings of Michael Platten (University of Heidelberg, Germany) on gliomas generated considerable excitement in the audience. He identified 2-hydroxyglutarate as a key metabolite generated in mutated gliomas. This molecule, formed by a mutated tricarboxylic acid (TCA) cycle enzyme, eventually orchestrates the induction of immune tolerance towards the tumor in immune cells of the tumor microenvironment. Most interestingly, Prof. Platten could demonstrate that pharmacological AHR inhibition resulted in a partial reversion of the intratumoral immune suppression and that it increased the efficacy of checkpoint blockade in a mouse model [13]. In comparison, David Sherr (Boston University, USA), in collaboration with Francisco Quintana (Brigham and Women’s Hospital, Harvard Medical School, USA), demonstrated that AHR knockout in oral cancer cells led to complete immune resistance to challenge with wild-type tumor cells. Indeed, this treatment resulted in a decrease in T cells expressing an exhausted phenotype and a decrease in cells expressing makers of immunosuppressive macrophages and granulocyte-like myeloid-derived suppressor cells (MDSC). Similar results were shown with a novel, small molecule AHR inhibitor.

**Figure 1:**
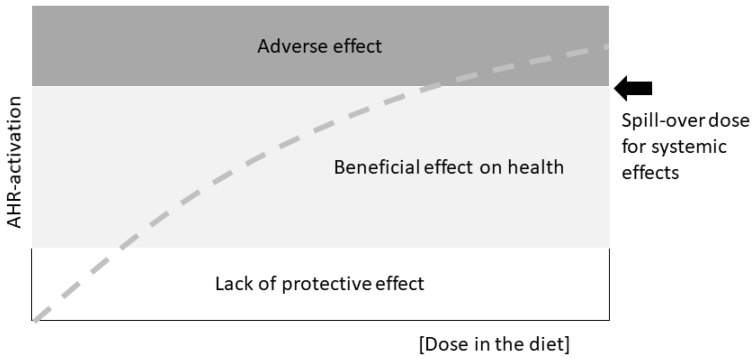
Graphical scheme of the possible health effects of a diet-provided AHR ligand at different doses. The thresholds will have to be determined experimentally. Not included are off-target effects or the possibility of effects of metabolites of the original agonist. Drawn after a slide presented by Gary Perdew at the AHR2018 meeting in Paris.

Looking at stem cells and developmental cues in adult, healthy tissue, Gary Perdew from the Pennsylvania State University, USA, reminded the audience of a fundamental caveat: AHR activity and downstream effects induced by various agonists fall into three categories: lack of protection, health benefits, and adverse effects (Figure 1). This is something important to keep in mind when discussing biological effects of AHR-activation or ligand treatment schemes, and firmly brings back the age-old toxicological tenet “the dose makes the poison”.

Dr. Perdew then demonstrated for gut enterocytes that the turnover rate of this constantly self-renewing epithelium depends on the activation of the AHR by diet-derived ligands. A diet rich in AHR-ligands links to the expression of genes responsible for enterocyte differentiation. Thus, AHR expression, which varies according to the region of the villi, alters enterocyte differentiation, cell turnover, and epithelial cell lineage fate. Other relevant sources of AHR-ligands such as diet and the gut microbiome were also topics of several other talks. “Broccoli” was the buzz-word often heard as a stand-in for a plant-based diet rich in AHR-ligands, and also as a trigger of sighs and smiles, depending on the culinary preferences in the audience.

Obesity is a global epidemic, and the underlying basis for many associated morbidities. Craig Tomlinson from the Norris Cotton Cancer Center at Dartmouth, USA, highlighted the role of the AHR in the regulation of metabolic functions (beside its regulatory role in tumor-disrupted metabolism). He reported that the tryptophan-derived AHR-ligand kynurenine stimulated AHR to enhance obesity in mice fed a “Western-Style” high fat diet. Weight gain was abolishable by an AHR-antagonist/partial AHR agonist, alpha-naphthoflavone. Prof. Tomlinson identified cytochrome P450 1B1, arachidonic acid, and peroxisome proliferator-activated receptor (PPAR)α as effectors down-stream of AHR activation [14]. In addition, Cornelis Elferink from the University of Texas, USA, extended the discussion on the AHR linkage with the occurrence of obesity/adiposity. In particular, he showed that AHR regulates the expression of fibroblast growth factor 21 (FGF21), a thermogenesis-promoting hormone. Deletion of the AHR in the liver increases FGF21 production, resulting in higher thermogenesis in the white adipose tissue of mice, which eventually lowers adiposity.

Disruption of the physiological production of AHR ligands by bacteria in the gut relates to inflammatory bowel disease and metabolic syndrome. Harry Sokol from the Sorbonne in Paris, France, showed that the ability of gut bacteria to produce AHR ligands is impaired in the context of inflammatory bowel disease. Levels of indole acetic acid (IAA), a tryptophan metabolite and AHR ligand are low in patients with Crohn’s disease and ulcerative colitis [15]. Similarly, high-fat diet-induced changes in the microbiota may drive metabolic syndrome, with bacteria producing decreased amounts of AHR ligands. This could contribute to mucosal inflammation and eventually systemic inflammation and down-regulation of the glucagon-like peptide 1 (GLP-1), a critical hormone that stimulates insulin secretion upon enteral glucose increase. Dr. Sokol also shared his data showing that feeding *Lactobacillus reuteri* or FICZ, a potent AHR agonist, can restore metabolic balance, and is beneficial for metabolic syndrome-associated health impairments. Of note, not all bacteria will “do the trick,” even in closely related bacterial species; thus, this effect resides in a bacterial species-specific metabolic capacity [16]. Marco Colonna (Washington University School of Medicine, St. Louis, USA), presented work relating to the immunological effects of *Lactobacillus reuteri*, focusing on a gut-specific subpopulation of intraepithelial T cells, which are both cluster of differentiation (CD)4 and CD8 positive. These cells have a regulatory function and can prevent local inflammation in a model of oral tolerance. However, they are absent in AHR-deficient mice, in germ free mice, and even in some commercial mouse-facilities [17]. Combining antibiotic treatment with 16S sequencing, his research group identified *L. reuteri* as the bacteria species that induces host development of these anti-inflammatory “double positive cells”. *L. reuteri* is relevant to AHR because it produces several AHR- ligands in addition to reuterin, a potent bactericide.

While these emerging findings support the importance of gut immune tolerance as a decisive feature of a healthy gut, the search is still on for AHR ligands that can be beneficial in therapy of inflammatory bowel disease, in particular ulcerative colitis, as well as for the metabolic syndrome. For instance, Dr. Takanori Kanai from Keio University, Japan, presented indoles, which are present at high concentrations in medicinal plants, such as *Indigo naturalis*, used in traditional medicine. He focused on indigo and indirubin, two AHR ligands capable of stimulating innate lymphoid cells (ILC) 3 and their IL-22 production. He reported that in multi-center studies the plant extract showed beneficial effects in ulcerative colitis patients over placebo controls and induced changes in dysbiosis after treatment. This is one example out of many where compounds in traditional medicine come into the limelight of AHR research [18].

Diet-related modulation is not restricted to the gut itself, but has systemic consequences. For example, Francisco Quintana from the Harvard Medical School, shared his work on the gut-brain-axis and beautifully showed the involvement of AHR in multiple sclerosis (MS), a disease in which the immune system attacks and destroys the myelin sheaths around nerves, resulting in paralysis. Astrocytes in the brain drive the low-grade inflammation in progressive MS. He reported that dietary tryptophan metabolites limit astrocyte pathogenicity in this context, and that AHR ligands activate the AHR in microglia and limit their intrinsic pathogenic activity via T cell growth factor (TGF)-α and vascular endothelium growth factor (VEGF)-β secretion. These and other findings he shared suggest that the AHR balances the beneficial-adverse interactions of astrocytes and microglia in the brain, an idea that may guide new therapies for MS. In a similar vein, Charbal Massaad, from the University Paris Descartes, France, investigated the role of the AHR in the function of Schwann cells, looking at its contribution to myelin development. In AHR-deficient mice, he correlated impaired locomotion with thinner myelin sheaths. Lack of AHR results in higher β-catenin levels, which are the drivers of myelin expression [19]. These latter findings raise important questions as to the role AHR-activation during development in shaping cellular and organ system function later in life. They also connect the AHR with the Wnt (wingless-related integration site) signaling pathway, a connection also made by several investigators [20]. B. Paige Lawrence, from the University of Rochester, New York, USA, reinforced the role of the AHR in organ (immune system) development. She followed the fate of CD4+ and CD8+ T cells of mice that had been exposed during development to the high-affinity AHR-ligand 2,3,7,8-tetrachlorodibenzo-p-dioxin, and demonstrated that the cells remain functionally impaired later in life. Further, she showed that changes in T cell responses to immune challenge could be transferred by transplanting immune progenitor cells or mature T cells from exposed mice into unexposed recipient mice, indicating that triggering AHR during development influences developmental programming. Such durable consequences of aberrant AHR-activation in development have far-reaching implications, and it will be necessary and helpful to study these for other vulnerable tissues as well, e.g., the nervous system. The underlying causes of dysregulated T cell functions may include changes in mitochondrial/energy threshold setting and pathway-determinations (discussed by Prof. Lawrence), as well as interference with long-range chromatin interactions (discussed by Sudin Bhattacharya from Michigan State University, USA), or mediated by miRNAs (Pilar Martin, Centro Nacional de Investigaciones Cardiovasculares Carlos III, Madrid, Spain). Thus, beyond the simple classical view that AHR drives change via effects by gene induction of AHR-responsive promoter bearing genes, these studies suggest that we need to view the AHR as part of a biochemical network that ensures development and differentiation, which facilitates adaption to environmental cues. In this context, it is worth mentioning that the AHR-repressor (AHRR), one of the early genes induced by the transcriptional AHR pathway, may also be involved in organ development. That is, there is growing evidence that the AHRR protein enables negative crosstalk of at least some AHR-mediated cellular events. In vivo studies are emerging, which will empirically test this connection [21]. Fabian Gondorf from Irmgard Förster´s group at the University of Bonn, Germany, showed that AHRR deficiency has significant impacts on cell metabolism, revealing increased oxidative phosphorylation in AHRR-deficient-macrophages, and resistance to adiposity development in AHRR-deficient KO mice. Given that AHR-deficient-mice also do not develop obesity when subjected to a high fat diet, it appears that AHRR could be an important target of the AHR signaling pathway, which contributes to the regulation of AHR-mediated metabolic control.

Aging and cellular senescence represent the flip side of the coin of prenatal development. Natascia Ventura, from the IUF and the University of Düsseldorf, Germany, introduced the nematode *Caenorhabditis elegans* as a novel model organism for AHR research. This lower eukaryote, although microscopic, is a multicellular organism composed of different tissues and permits powerful genetic, aging and toxicology studies in vivo. Dr. Ventura showed that, in *C. elegans*, a mutated AHR delays aging and age-associated pathologies in an evolutionarily conserved manner [22]. She reported that AHR-activating ligands known from mouse and man also modulated AHR-dependent phenotypes in *C. elegans*, albeit the mechanisms are still unknown. Currently, the group is in fact investigating whether *C. elegans* AHR activity is modulated in a ligand-binding-dependent or -independent manner. Nonetheless, as the AHR is highly conserved, the use of *C. elegans* and other non-mammalian model organisms will certainly expand our knowledge of AHR‘s role in biological processes.

Finally, most of the studies presented at the 2018 AHR meeting either demonstrated or implied the therapeutic potential of AHR-ligands (agonists and antagonists) in disease settings. Indeed, the myriad of AHR-mediated outcomes in various organs is intriguing and suggests several applications for AHR-specific modulators [23]. Even so, the breadth of AHR activity and the context (organ, ligand)-specific effects suggests caution when using AHR agonists or antagonists because of the potential on-target side effects. Two general sets of results mitigate against the argument that AHR modulators would adversely affect normal tissue in the disease setting: (1) A similar or perhaps greater level of concern was exhibited for many now-proven effective therapeutics that target over-expressed or over-active molecules, e.g., Letrozole, an aromatase inhibitor used to treat hormone-driven cancers [24,25], Bortezomib, a proteasome inhibitor used to treat multiple myeloma [26,27] and most kinase inhibitors used for several types of cancers; (2) To date, no “show stopper” side effects have been seen with AHR modulators in vivo. For example, Mary Walker (University of New Mexico, USA) showed work done together with scientists from Teva Pharmaceuticals, Netanya, Israel. She demonstrated that Laquinimod, an AHR agonist, may be an effective drug for multiple sclerosis and, in Phase III clinical trials exhibited (thus far) acceptable levels of side effects despite some regulators’ concerns about “dioxin-like toxicities”. A group including Michael Platten, David Sherr, Karen McGovern (Kyntherapeutics, Cambridge, MA, USA), and Ulrich Deuschle (Phenex Pharmaceuticals, Ludwigshafen, Germany) have used AHR inhibitors to enhance immunity and/or suppress tumor growth with no reported side effects in animal models. Francisco Quintana demonstrated that naturally occurring microbial or dietary AHR ligands limit experimental allergic encephalomyelitis (EAE) without overt side effects. Barbara Postal (UMRS/INSERM 1138, Centre de Recherche des Cordeliers) demonstrated that AHR ligands could inhibit gut inflammation without overt side effects. Francesca Fallarino (University of Perugia, Italy) showed that in mice the engagement of the AHR by specific tryptophan derivates may be very beneficial in controlling unwanted antibody formation upon Factor VIII treatment against hemophilia. Takanori Kanai demonstrated that indigo, an AHR agonist, reduces ulcerative colitis with no overt side effects. And in closing a circle from the mediators of environmental pollutant toxicity to relatively benign therapeutic, Ellen van den Bogaard, Radboud University Medical Center, Nijmegen, The Netherlands, showed data where the polycyclic aromatic hydrocarbon (PAH) mixture in coal tar activates the AHR and reduces inflammation of psoriasis and atopic skin dermatitis without the AHR-mediated toxicity of a classic PAH. In addition, other structurally diverse AHR ligands have similar effects, which opens avenues for the therapeutic use of AHR ligands in inflammatory skin diseases. In short, within any given disease, tissue, or AHR ligand context, it seems, at least thus far, that a wide enough therapeutic window exists to expect AHR modulators to have some beneficial effect without significant on-target side effects.

## 3. Conclusions

The 4th AHR-conference showed that research on the AHR is developing fast and is leading us towards new areas beyond toxicology and immunology. In particular, the meeting highlighted the role of the AHR and its associated signaling paths in developmental, metabolic, immunologic, and pathologic processes. The rapidly increasing understanding of the AHR is on track to enable therapeutic and preventive applications, both in the areas of pharmacology and nutrition. AHR modulation may well prove to be a powerful treatment for cancer, skin diseases, neurological and metabolic diseases.

## Abbreviations

AHRR	Aryl hydrocarbon receptor repressor
CD	Cluster of differentiation
MS	Multiple sclerosis
PAH	Polycyclic aromatic hydrocarbons
